# Are the Closely Related *Cobetia* Strains of Different Species?

**DOI:** 10.3390/molecules26030690

**Published:** 2021-01-28

**Authors:** Yulia Noskova, Aleksandra Seitkalieva, Olga Nedashkovskaya, Liudmila Shevchenko, Liudmila Tekutyeva, Oksana Son, Larissa Balabanova

**Affiliations:** 1 Laboratory of Marine Biochemistry, G.B. Elyakov Pacific Institute of Bioorganic Chemistry, Far Eastern Branch, The Russian Academy of Sciences, 690022 Vladivostok, Russia; noskovaiulia@yandex.ru (Y.N.); sasha0788@inbox.ru (A.S.); oned2004@mail.ru (O.N.); lshev@piboc.dvo.ru (L.S.); 2 Basic Department of Bioeconomy and Food Security, School of Economics and Management, Far Eastern Federal University, 690090 Vladivostok, Russia; tekuteva.la@dvfu.ru (L.T.); oksana_son@bk.ru (O.S.)

**Keywords:** *Cobetia amphilecti*, *Cobetia litoralis*, *Cobetia pacifica*, *Cobetia marina*, *Cobetia crustatorum*, identification markers, alkaline phosphatase PhoA

## Abstract

Marine bacteria of the genus *Cobetia,* which are promising sources of unique enzymes and secondary metabolites, were found to be complicatedly identified both by phenotypic indicators due to their ecophysiology diversity and 16S rRNA sequences because of their high homology. Therefore, searching for the additional methods for the species identification of *Cobetia* isolates is significant. The species-specific coding sequences for the enzymes of each functional category and different structural families were applied as additional molecular markers. The 13 closely related *Cobetia* isolates, collected in the Pacific Ocean from various habitats, were differentiated by the species-specific PCR patterns. An alkaline phosphatase PhoA seems to be a highly specific marker for *C. amphilecti.* However, the issue of *C. amphilecti* and *C. litoralis*, as well as *C. marina* and *C. pacifica,* belonging to the same or different species remains open.

## 1. Introduction

Bacteria of the genus *Cobetia* are Gram-negative, aerobic and halotolerant and belong to the *Halomonadaceae* family. For the first time, a bacterium of the genus *Cobetia* was described in 1970 by Cobet et al. [1] and was originally assigned to the species *Arthrobacter marinus* sp. nov. In further studies, it was assigned to various genera, such as *Pseudomonas* [2], *Deleya* [3], and *Halomonas* [4]. Then, based on an analysis of the 23S and 16S rRNA sequences and amending the description of the species *Halomonas marina*, including new features, the authors [5] proposed assigning it to a new genus, *Cobetia* gen. nov., within the *Halomonadaceae* family. The type species is *Cobetia marina*, with the type strain LMG 2217 (= CIP 104,765 = IAM 14,107 = CECT 4278 = DSM 4741 = NCIMB 1877 = CCUG 49,558 = NBRC 102,605 = JCM 21,022 = ATCC 25374).

At present, a few species are characterized for marine bacteria of the genus *Cobetia*, namely: *C. marina* [5], *C. crustatorum* [6], *C. amphilecti* [7]*, C. pacifica* [7], and *C. litoralis* [7]. The whole genome sequence analysis is presented only for three strains of the genus *Cobetia*, including a type strain *C. marina* JCM 21022^T^ [8], and the non-type strains *C. amphilecti* KMM 296 (formerly *C. marina* KMM 296) [9] and *Cobetia* sp. cqz5-12 [10]. At the same time, more and more information appears about *Cobetia* isolates, which are promising sources of unique enzymes and secondary metabolites degrading oil [11], bacterial biofilms [12], alginate [10,13], and phenol [14]. However, their species identification are complicated due to the high level of identity for their 16S rRNA genes and the absence of whole genome sequences for the type strains of known species, excluding from *C. marina* JCM 21022^T^ [8]. The 16S rRNA gene usually is highly specific to each bacterial species that makes it a good target for identification of both environmental and clinical bacterial isolates [15,16,17]. However, the 16S rRNA sequences were found to be indistinguishable for a few species [18]. The usage of 16S rRNA gene as a marker is limited for the closely related species, which have a high percentage of the sequence similarity and lack enough variations. Thus, a conventional method of 16S rRNA phylogeny has often failed to correctly identify *Vibrio* species [19]. Similarly, we found that the *Cobetia* species have 99–100% identity of their compared 16S rRNA sequences. In this regard, searching for the additional molecular markers and/or methods for the species identification of the *Cobetia* isolates is especially relevant.

In this work, we have applied the genome-based found species-specific coding sequences for the essential enzymes from each functional category and different structural families, such as nucleases, proteases, phosphatases, and phospholipase, as the additional molecular markers in the polymerase chain reaction (PCR)-based method, to differentiate the species of the closely related isolates of the marine bacteria *Cobetia,* collected in the Pacific Ocean from various habitats. In general, we have raised the issue of the possible species reclassification, due to their highly homologous 16S rRNA sequences, PCR-patterns with the use of the additional molecular markers, which were suggested here, and a high similarity between two whole genome sequences of the closely related non-type strains.

## 2. Results and Discussion

Thirteen strains of the marine bacteria, isolated from coastal seawater and sediments from the Sea of Japan, marine invertebrates, the mussel *Crenomytilus grayanus* from the Sea of Japan and the deep-water sponge *Esperiopsis digitate* from the Sea of Okhotsk, and the red algae *Ahnfeltia tobuchiensis* from the Sea of Okhotsk, which are deposited in the Collection of Marine Microorganisms (KMM, G.B. Elyakov Pacific Institute of Bioorganic Chemistry, Far Eastern Branch, Russian Academy of Sciences, http://www.piboc.dvo.ru/), were assigned to the genus *Cobetia* by physiological, biochemical and molecular genetic parameters, using sequencing and phylogenetic analysis of their 16S rRNA genes (Table 1). However, the strain-specific metabolic versatility and ecophysiological diversity of these *Cobetia* isolates could not allow distinguishing between their species [9,20,21]. Thus, our study showed that 7 from 13 strains have 99.86–100% identity of the 16S rRNA genes simultaneously to two type strains *C. marina* LMG 2217^T^ (JCM 21022^T^) and *C. pacifica* KMM 3879^T^ (NRIC 0813^T^), one strain has 100% identity to the type strain *C. crustatorum* JCM 15644^T^, and three strains have 100% identity to the type strain *C. amphilecti* KMM 1561^T^ (NRIC 0815^T^) (Table 1). The strain *Cobetia* sp. 2AS (KMM 7514), isolated from a coastal seawater, showed 99.93 and 99.86% similarities with *C. amphilecti* KMM 1561^T^ and *C. litoralis* KMM 3880^T^, respectively (Table 1). From four independent replicates, half of the 16S rRNA DNA samples from the clones of the strain *Cobetia* sp. 29-18-1 (KMM 7000), isolated from the sponge, were of 100% identity with the type strain *C. amphilecti* KMM 1561^T^ (NRIC 0815^T^), and the other half had 100% identity with the 16S rRNA gene of the type strain *C. litoralis* KMM 3880^T^ (NRIC 0814^T^) (Table 1). Therefore, the EzBioCloud identification, based on the use of 16S rRNA gene sequences of the type strains [22], showed only one of the identical reference records in its database (Table S1). Furthermore, the results of similarity calculation by EzBioCloud 16S database showed that the 16S rRNA genes are completely identical for *C. marina* JCM 21022^T^ and *C. pacifica* KMM 3879^T^ (100% identity), and the only single nucleotide polymorphism (99.93% identity) is between the type strains *C. amphilecti* KMM 1561^T^ and *C. litoralis* KMM 3880^T^ (Tables S2 and S3).

The comparative genomics of *Cobetia* isolates is also impossible due to the absence of the type strains’ whole genome sequences to date except for *C. marina* JCM 21022^T^ [8]. Moreover, the next-generation sequencing (NGS) solutions cannot always allow resolving this problem quickly without detailed bioinformatics analysis. Thus, the whole genome shotgun sequencing for the strain *Cobetia* sp. 2AS1, KMM 7005 (GenBank: JADAZN000000000.1) has led to the loss of its complete 16S rRNA gene, and consequently, indicated the same similarity (100%) of its partial sequence to both *C. amphilecti* KMM 1561^T^ and *C. litoralis* KMM 3880^T^ with the use of EzBioCloud identification system “Genome-based ID” [22].

**Figure 1:**
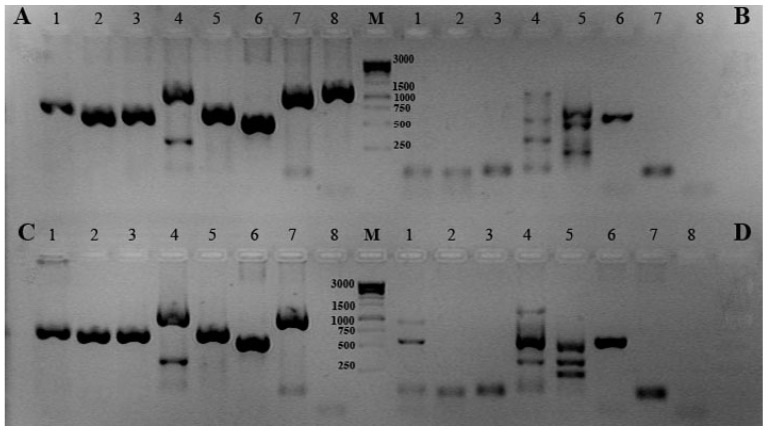
Gel-electrophoresis of PCR products of the new molecular markers: (**A**) ***C. amphilecti* KMM 1561^T^**, *C. amphilecti* KMM 296 and KMM 7516; (**B**) ***C. marina* LMG 2217^T^** and KMM 6284; (**C**) ***C. litoralis* KMM 3880^T^**, KMM 7000, KMM 7005 and KMM 7514; (**D**) ***C. pacifica* KMM 3879^T^**, KMM 6731, 7505, 7508, 7515, 6816, and 6818. **Lane numbers: 1**—DNA/RNA non-specific endonuclease precursor; **2**—DNA/RNA non-specific endonuclease; **3**—EEP-like (DNaseI-like) nuclease; **4**—alkaline phosphatase/phosphodiesterase PhoD; **5**—phospholipase A; **6**—ATP-dependent protease Clp; **7**—periplasmic serine peptidase Do/DeqQ; **8**—CmAP-like alkaline phosphatase PhoA; **M**—1 kb DNA ladder marker (Evrogen).

**Figure 2:**
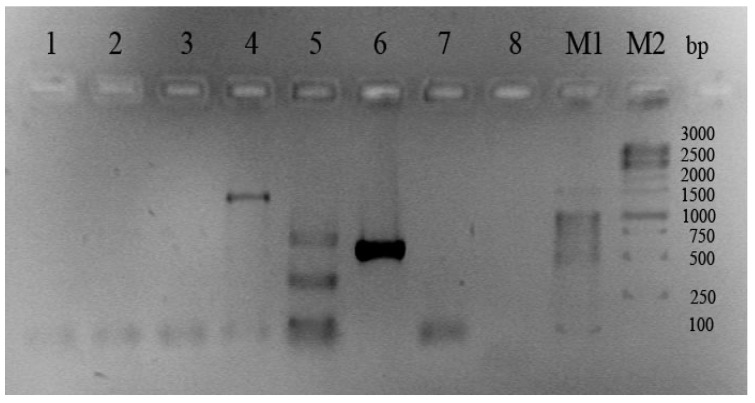
Gel electrophoresis of PCR products of the new molecular markers in the strains *C. crustatorum* JCM 15644^T^ and *C. crustatorum* KMM 6817. Lane numbers: **1**—DNA/RNA non-specific endonuclease precursor; **2**—DNA/RNA non-specific endonuclease; **3**—EEP-like (DNaseI-like) nuclease; **4**—alkaline phosphatase/phosphodiesterase PhoD; **5**—phospholipase A; **6**—ATP-dependent protease Clp; **7**—periplasmic serine peptidase Do/DeqQ; **8**—CmAP-like alkaline phosphatase PhoA; **M**—1 kb DNA ladder marker (Evrogen).

To clarify the species identity of this coastal seawater isolate *Cobetia* sp. 2AS1 (KMM 7005), we have tried to obtain PCR-products, using the sequences of structural genes encoding for the key metabolic hydrolases of the non-type strain *C. amphilecti* KMM 296 associated with a mussel, which were selected for the functional genomic studies for this species (GenBank: JQJA00000000.1). Putative coding DNA sequences (CDSs) for the following enzymes were selected as identification markers: alkaline phosphatases of the structural families PhoA (DQ435608) and PhoD (WP_043333989); EEP-like (DNaseI-like) nuclease (WP_084589364) and DNA/RNA non-specific (S1-like) endonuclease (WP_043334786), ATP-dependent protease Clp (KGA03297), phospholipase A (WP_084589432), and periplasmic serine peptidase with thrypsin-like peptidase domain of the Do/DeqQ family (KGA03014). The Do/DeqQ family serine peptidase has a chaperone function at low temperatures and proteolytic activity at elevated temperatures, thus protecting bacteria from thermal and other stresses [23]. Clp proteases are involved in a number of cellular processes, such as degradation of misfolded proteins, regulation of short-lived proteins and housekeeping removal of dysfunctional proteins, the control of cell growth, and targeting DNA-binding protein from starved cells [23]. The large EEP (exonuclease/endonuclease/phosphatase) domain superfamily (structural family: cl00490) includes a diverse set of proteins, including the ExoIII family of apurinic/apyrimidinic (AP) endonucleases, inositol polyphosphate 5-phosphatases (INPP5), and deoxyribonuclease I (DNaseI), which share a common catalytic mechanism of cleaving phosphodiester bonds, with the substrates range from nucleic acids to phospholipids and, probably, proteins [23]. The DNA/RNA endonuclease belonging to the structural cl00089: NUC superfamily can non-specifically cleave both double- and single-stranded DNA and RNA, whose domain may be present in phosphodiesterases [23]. Thus, all these enzymes are fundamental for bacteria survival [24,25,26,27,28,29]. If the strains *Cobetia* sp. 2AS1 (KMM 7005) and *C. amphilecti* KMM 296 are of the same species, they should have a high level of their CDS similarity and the same pattern of the presence and distribution of the PCR products by electrophoresis. In addition, the same PCR primers were also applied to all type strains and new isolates belonging to the genus *Cobetia* (Figure 1 and Figure 2).

Figure 1 shows the results of gel-electrophoresis of the PCR products for the type strains of the *Cobetia* species: *C. amphilecti* NRIC 0815^T^ (KMM 1561^T^) (A), *C. marina* LMG 2217^T^ (B), *C. litoralis* NRIC 0814^T^ (KMM 3880^T^) (C), *C. pacifica* NRIC 0813^T^ (KMM 3879^T^) (D), and *C. crustatorum* JCM 15644^T^ (Figure 1 and Figure 2). The type strain of each species is characterized by an individual distribution of the PCR products that could allow classifying the new *Cobetia* isolates by these patterns (Figure 1 and Figure 2). Because of the PCR-based method for the molecular differentiation, using the gene-specific primers and the genomic DNA of the strains under study, they can be divided into five groups belonging to the five species of the genus *Cobetia*. The type strain *C. amphilecti* NRIC 0815^T^ (KMM 1561^T^) and the strains *C. amphilecti* KMM 296 and KMM 7516 had an identical pattern of distribution for the PCR products of all new eight molecular markers, which indicate their 100% homology accordingly to the results of 16S rRNA analysis (Figure 1A, Table 1 and Table S1).

The next group of the strains, with an identical PCR pattern, includes two *C. marina* strains: LMG 2217^T^ and KMM 6284 (Figure 1B), which expectedly lost most PCR products due to the more distant relation to *C. amphilecti* by both their 16S rRNA genes and whole genome sequences [8,9].

Remarkably, the strain *Cobetia* sp. KMM 7000, and two strains KMM 7514 (2AS) and KMM 7005 (2AS1), which have higher homology by 16S rRNA sequences with the species *C. amphilecti* (Table 1 and Table S1), showed the complete identity of the PCR product distribution with the type strain *C. litoralis* KMM 3880^T^ (Figure 1C). However, both type strains of *C. amphilecti* and *C. litoralis* have been found to possess highly homologous CDSs for all the predicted hydrolases (Figure 1A,C), used here as the molecular markers, except for the well-studied highly active PhoA-like alkaline phosphatase CmAP (Figure 1A,C, lanes **8**) [30]. The question arose as to how close these strains can be to each other, taking into account the fact that their physiological parameters and the results of DNA:DNA hybridization allowed them to be attributed to different biological species [7]. A comparative analysis of the whole genome sequence of *Cobetia* sp. 2AS1 (KMM 7005) (GenBank: JADAZN000000000.1), with the use of the SEED Viewer at the Rapid Annotation using Subsytems Technology (RAST) server [31], confirmed that the most CDSs for the enzymes, used as the marker genes (Table S4; column **B**: 2373, 3075, 2144, 1612, 617, 1748, 322), are similar with those of *C. amphilecti* KMM 296 by 99.32–99.64%, except for phospholipase A1 (97.14%) and protease Cpl (100%). However, an alkaline phosphatase PhoA, structurally similar to the alkaline phosphatase CmAP from *C. amphilecti* KMM 296, is absent in *Cobetia* sp. 2AS1 (KMM 7005) (Table S4). A putative orthologue (alkaline phosphatase EC 3.1.3.1), which should carry a similar function, showed only 38.39% identity with CmAP (Table S4, column B: 1612). Generally, 94.84% from 3253 CDSs of *Cobetia* sp. KMM 7005 (GenBank: JADAZN000000000.1) showed an average similarity of 83.5% with CDSs of *C. amphilecti* KMM 296 (GenBank: JQJA00000000.1) that is approximately correspondent to 88% average nucleotide identity (ANI) of their genomes (Table S4).

The high percentage of 16S rRNA genes’ (99.93–100%) and whole genomes’ (88%) identities may mean that the strains of *C. amphilecti* and *C. litoralis* belong to the same species, but currently they are undergoing significant phenotypic and genotypic divergence because of adaptive evolution [32]. Possibly, the highly active alkaline phosphatase PhoA was acquired by the cosmopolite *Cobetia* strains during their trying colonization of an invertebrate digestive tract due to the putatively significant role of the enzyme in the relationship (symbiotic or pathogenic) between marine habitants, such as *C. amphilecti* KMM 296 and the mussel *C. grayanus* or *C. amphilecti* KMM 1561^T^, and the eponymous sponge *Amphilectus digitatus* [7,9,20,24]. Meanwhile, their closely related strains of *C. litoralis*, including the type strain KMM 3880^T^, were isolated predominantly from coastal sediments, therefore, they may not need such enzymatically active and specific alkaline phosphatase as CmAP [7,30]. The 16S rRNA heterogeneity of *C. litoralis* KMM 7000 may be an additional evidence of the species divergence due to the adaptation to colonization of marine invertebrates, which are the predominant habitats of the closely related strains of the species *C. amphilecti*, including KMM 296 and KMM 1561^T^ [7,20,21]. Thus, a squid-vibrio symbiosis is feasible by modulation of the bacterial symbiont lipid A signaling by the host alkaline phosphatases facilitating its colonization of the juvenal squid’s light organ [33]. The urgent need for mineralization and repair of the invertebrate’s exoskeleton can also be a key factor in symbiosis with a carrier of a highly efficient nonspecific phosphatase like CmAP [9,30,34]. However, the conclusion should be drawn only after sequencing the whole genomes of the type strains of *Cobetia* and elucidation of biological functions of their species- and strain-specific genes and proteins. In addition, such a high adaptability and metabolic versatility in various environmental conditions requires investigating the possible contribution of the bacterium to the toxicity or pathogenicity of shellfish, particularly, for the humans consuming them raw.

A similar situation may be with other closely related species *C. marina* and *C. pacifica*. The largest group of our isolates, assigned to the species *C. marina/C. pacifica* (KMM 7508, 7515, 6816, 6818, 7505 and 6731), have 100% identity by 16S rRNA with both species, but their results from the suggested PCR-based method correspond to the species-specific pattern inherent for the type strain *C. pacifica* KMM 3879^T^ (Figure 1D, Table 1 and Table S1). The dominant differences between these species were in the lanes 1, 4, and 5, indicating the differences in their PCR-targeted sequences (Figure 1B,D).

Finally, *C. crustatorum* KMM 6817 and the type strain *C. crustatorum* JCM 15644^T^ significantly differ from other groups of the *Cobetia* isolates in the number and location of the bands of PCR products that correspond to the 16S rRNA analysis results (Figure 2, Table 1 and Tables S1–S3).

Thus, the suggested molecular markers used in the PCR-based method allowed distinguishing the isolates of *C. marina* and *C. amphilecti* from the isolates of *C. pacifica* and *C. litoralis*, respectively. From seven isolates of indistinguishable 16S rRNA sequences, only one from the red algae seeds was identified as *C. marina* (KMM 6284) and the others were of *C. pacifica* (KMM 7508, 7515, 6816, 6818, 7505, and 6731), isolated from algae and coastal seawater (Table 1). Five isolates of *C. amphilecti* and *C. litoralis*, indistinguishable by 16S rRNA analysis, were assigned as two *C. amphilecti* (KMM 296 from the mussel and KMM 7516 from coastal seawater) and three *C. litoralis* (KMM 7005, KMM 7514 from sediments and KMM 7000 from the sponge). The strain *Cobetia* sp. KMM 6817 was easily assigned to the *C. crustatorum* species according to the results of both methods of analysis, which proves the validity of the suggested molecular markers (Table 1).

According to the results of the *Cobetia* species identification at this stage of investigation, there is a tendency for the predominant association of the Pacific Ocean populations of *C. pacifica*, *C. amphilecti* and *C. litoralis* with algae, invertebrates and sediments or coastal water, respectively (Table 1). However, to confirm these observations, a more extensive search for isolates from different habitats should be carried out.

## 3. Materials and Methods

### 3.1. Isolation of the Strains Belonging to the Genus Cobetia

Bacterial strains were isolated from the different marine environments, including coastal waters, sediments, seaweeds and animals, using the standard dilution-plating method. Strains KMM 6284 and KMM 6731 were directly isolated from the red alga *Ahnfeltia tobuchiensis* collected near Island Paramushir, Kuril Isles, the Sea of Okhotsk, and the Pacific Ocean. Strains KMM 6816, 6817 and 6818 were isolated from the same alga after its long-term continuous cultivation in the natural seawater for 6 months. Strain KMM 7000 was recovered from the sponge *Esperiopsis digitata,* collected near Island Sakhalin, Piltun Bay, the Sea of Okhotsk, from depth of 107 m by plating on medium A containing (L^−1^): 2 g Bacto peptone (Difco), 2 g Bacto yeast extract (Difco), 1 g casein hydrolysate, 0.2 g KH_2_PO_4_ and 0.1 g ferric ammonium citrate prepared with 50% (*v*/*v*) natural sea water and 50% (*v*/*v*) distilled water. For strains isolation, 0.1 mL of tissue homogenate was transferred onto plates of marine agar 2216 (MA; Difco) (strains KMM 6284, 6731, 6816, 6817, and 6818) or medium A or medium B. After primary isolation and purification, the strains were cultivated at 28 °C on the same medium and stored at –80 °C in marine broth (Difco) supplemented with 20% (*v*/*v*) glycerol. Strains KMM 7516, 7505, 7515 and 7508, and 7514 and 7005 were isolated from coastal seawater and sediment samples, respectively, collected from Vostok Bay, the Sea of Japan, by plating on medium B, containing: 1 g (NH_4_)_2_SO_4_, 0.2 g MgSO_4_ 7H_2_O, 1 g K_2_HPO_4_, 1 g KH_2_PO_4_, 0.02 g CaCl_2_, 0.05 g FeCl_3_, and 20 g NaCl in 1000 mL distilled water, with the addition of 1 mL per liter of sterile crude oil to the prepared medium. Strain *Cobetia* sp. KMM 296, previously identified as *Deleya marina* by Ivanova et al. [35], was received from the Collection of Marine Microorganisms (KMM, Russia). The type strains of the recognized species *Cobetia amphilecti* KMM 1561^T^ (NRIC 0815^T^), *Cobetia litoralis* KMM 3880^T^ (NRIC 0814^T^), *Cobetia pacifica* KMM 3879^T^ (NRIC 0813^T^), and *Cobetia marina* LMG 2217^T^ were used as reference strains and kindly provided to us by the NODAI Culture Collection Center, (University of Agriculture, Tokyo, Japan) and from the BCCM/LMG Bacteria Collection (Universiteit Gent, Gent, Belgium), respectively [7]. The reference type strain *C. crustatorum* JCM 15644^T^ was kindly provided by the Japan Collection of Microorganisms (RIKEN BioResource Centre (BRC), Tsukuba, Japan). 

### 3.2. Isolation and Analysis of DNA from the Strains

All strains of marine bacteria were cultivated in Petri dishes on sterile agar LB medium of the following composition (g/L): bacto-tryptone—10; yeast extract—5; NaCl—5; agar-agar—15; distilled water—0.98 l; the pH of the medium is 7.7. Five passages were made for each type of bacterium. Then, DNA was isolated from the grown colonies using the PureLink Genomic DNA Mini Kit protocol (Invitrogen). The isolated bacterial DNA was used to carry out a polymerase chain reaction (PCR), with universal primers to 16S rRNA sequences (BF/20: 5′-AGAGTTTGATCMTGGCTCA -3′; BR2/22: 5′- TACGGTTACCTTGTTACGACTT -3′) or the primers corresponding to the coding DNA sequences (CDSs) for housekeeping enzymes used in this work as molecular markers for the *Cobetia* species identification (Table 2).

Reaction conditions for PCR of 16S rRNA sequence in DNA amplifier C1000TM Thermal Cycler (Bio-Rad Laboratories, Inc., California, USA) or Mastercycler gradient (Eppendorf, Hamburg, Germany): 10× buffer for polymerase, 50× mixture of Encyclo polymerases (“Encyclo PCR kit”, Evrogen, Moscow), 50× mixture of dNTP (10 mM each), mixture of forward and reverse primers (5 µM each), and 20 ng DNA of a bacterial clone. The amplification process consists of the following stages: 30 PCR cycles × (15 s—95 °C; 30 s—55 °C; 1 min 30 s—72 °C), then incubation at 72 °C for 5 min. After amplification, PCR products were used for sequencing. The PCR products from each clone of each strain (four replicates) were sequenced and verified with the use of an ABI Prism 3130xl sequencer and Chromas program (version 2.5.1), respectively. Homology searches were performed against EzBioCloud 16S database using the Blast program to find sequences that provide significant alignment [22].

Reaction conditions for marker genes (Table 2) in DNA amplifier C1000TM Thermal Cycler (Bio-Rad Laboratories, Inc., California, USA) or Mastercycler gradient (Eppendorf, Hamburg, Germany): 10× Encyclo buffer, 50× Encyclo polymerase mixture (Encyclo PCR kit, Evrogen, Moscow), 50× dNTP mixture (10 mM each), a mixture of forward and reverse primers (5 µM each), and 20 ng DNA of a bacterial clone. The amplification process consisted of the following stages: 38 PCR cycles x (2 min—95 °C; 15 s—95 °C; 1 min 40 s—72 °C). After amplification, the PCR products from each clone of each strain (four replicates) were separated by gel electrophoresis in a 1% agarose gel stained with ethidium bromide. The PCR product visualization and documentation were performed with Herolab imaging system (Herolab GmbH Lab., Wiesloch, Germany). The PCR products for the species-specific coding sequencing regions of *C. amphilecti* and *C. litoralis* were confirmed by sequencing as described above.

Comparative analysis of the marker genes and whole genome sequences of *Cobetia* sp. 2AS1 (GenBank: JADAZN000000000.1) and *C. amphilecti* KMM 296 (GenBank: JQJA00000000.1) was carried out by the SEED Viewer at the RAST server [31].

## 4. Conclusions

The gene-specific oligonucleotides corresponding to the coding DNA sequences for the enzymes responsible for the vital bacterial cell functions, such as EEP-like and DNA/RNA nonspecific nucleases, alkaline phosphatases PhoA and PhoD, proteases Cpl and Do/DeqQ family, and phospholipase A1, may be used for rapid molecular differentiation of the closely related species of the marine bacteria *Cobetia* in addition to the traditional 16S rRNA assay. Furthermore, the highly active alkaline phosphatase CmAP of the structural family PhoA is a highly specific marker for the species *C. amphilecti*, probably indicating the adaptability to the host–microbe relationships. However, the genus needs further study, including verification of the suggested molecular markers with the use of a higher number of isolates to develop the MLST-based method of identification. Primarily, comparative genomics of the type strains of *Cobetia* should be carried out to a final decision about the valid interspecies phylogeny of the genus, particularly towards the closely related species (*C. amphilecti* and *C. litoralis*; *C. marina* and *C. pacifica*), as well as about their genetic drivers and causes of such divergent evolution within the genus.

## Figures and Tables

**Table 1:** The species identification of *Cobetia* isolates from the Pacific Ocean.

Isolate	Isolation Source	Collection Number, KMM *	Results of Identification		16S rRNA Genbank ID
			16S rRNA, % Identity	PCR-Based Method	
*Cobetia* sp. 1AS1	Coastal seawater, Vostok Bay, Sea of Japan	KMM 7516	*C. amphilecti, 100%*	*C. amphilecti*	MW332480
*C. amphilecti* KMM 296	Coelomic liquid, mussel *Crenomytilus grayanus*	KMM 296	*C. amphilecti, 100%*	*C. amphilecti*	NZ_JQJA01000078
*Cobetia* sp. 29-18-1	Sponge *Esperiopsis digitata,* Sea of Okhotsk, Is. Sakhalin, Piltun bay, 107 m.	KMM 7000	*C. amphilecti, 100%*/*C. litoralis, 100%*	*C. litoralis*	MW332487
*Cobetia* sp. 2AS	Sediments, Vostok Bay, Sea of Japan	KMM 7514	*C. amphilecti, 99.93%*/*C. litoralis 99.86%*	*C. litoralis*	MW332483
*Cobetia* sp. 2AS1	Sediments, Vostok Bay, Sea of Japan	KMM 7005	*C. amphilecti, 100%*	*C. litoralis*	MW332484
*Cobetia* sp. 41-10Alg46	The red algae *Ahnfeltia tobuchiensis,* collected near Is. Paramushir, Kuril Isles, Sea of Okhotsk	KMM 6284	*C. marina*/*C. pacifica, 100%*	*C. marina*	MK587632
*Cobetia* sp. 2S	Coastal seawater, Vostok Bay, Sea of Japan	KMM 7508	*C. marina*/*C. pacifica, 99.86%*	*C. pacifica*	MW332481
*Cobetia* sp. 3AS	Coastal seawater, Vostok Bay, Sea of Japan	KMM 7515	*C. marina*/*C. pacifica, 99.86%*	*C. pacifica*	MW332482
*Cobetia* sp. 11Alg1	The red algae *A. tobuchiensis* (long-time cultivated), collected near Island Paramushir, Kuril Isles, Sea of Okhotsk	KMM 6816	*C. marina*/*C. pacifica, 100%*	*C. pacifica*	MW332485
*Cobetia* sp. 11Alg14	The red algae *A. tobuchiensis* (long-time cultivated), collected near Island Paramushir, Kuril Isles, the Sea of Okhotsk	KMM 6818	*C. marina*/*C. pacifica, 100%*	*C. pacifica*	MW332486
*Cobetia* sp. 3AK	Coastal seawater, Vostok Bay, Sea of Japan	KMM 7505	*C. marina*/*C. pacifica, 100%*	*C. pacifica*	MW332488
*Cobetia* sp. 41-10Alg146	The red alga A. tobuchiensis, collected near Is. Paramushir, Kuril Isles, Sea of Okhotsk	KMM 6731	*C. marina*/*C. pacifica, 100%*	*C. pacifica*	KC247358
*Cobetia* sp. 11Alg4	The red algae *A. tobuchiensis* (long-time cultivated), collected near Is. Paramushir, Kuril Isles, Sea of Okhotsk	KMM 6817	*C. crustatorum, 100%*	*C. crustatorum*	MW332489

* KMM—Collection of Marine Microorganisms, G.B. Elyakov Pacific Institute of Bioorganic Chemistry, Far Eastern Branch, Russian Academy of Sciences, http://www.piboc.dvo.ru/.

**Table 2:** Oligonucleotides for molecular differentiation of *Cobetia* species.

Name	Sequence	Molecular Marker *	Reference **
1CmNucF	5′–TATACCATGGACGATATTCGCTCGGCCGGCCGCAA-3′	DNA/RNA non-specific endonuclease precursor (1) and without leader peptide (2)	WP_043334786
CmNucR	5′–TATAGAGCTCTCAGTAACGTGATGGCGTACGACTG-3′		
2CmNucF	5′-TATACCATGGTATGGCAGGAGCGCGACTACCAGCA-3′		
CmNucR	5′–TATAGAGCTCTCAGTAACGTGATGGCGTACGACTG -3′		
CmEEPf	5′–TATACCATGGGACTCGACGAGACGGCACCTCCCCT -3′	exonuclease/ endonuclease/phosphatase (EEP)	WP_084589364
CmEEPr	5′–TATAGAGCTCTTATGCTAGCCCGATCGCCTTGCGGCA-3′		
CmPhoDf	5′–TATACCATGGAAGGACGGCGCCCGCGCATGCCCTC-3′	alkaline phosphatase/phosphodiesterase PhoD	WP_043333989
CmPhoDr	5′–TATAGAGCTCTTAGACACTGGCGGCGGCGGGGGTC-3′		
CmPLA_f	5′–TATACCATGGTACTCGATGAAAGCCTGGCCCAGCA-3′	phospholipase A	WP_084589432
CmPLA_r	5′–TATAGAGCTCTTAGGTCTCTGGCGAGCCGGCGAAG-3′		
Tryp_F	5′–TATACCATGGTACGTGAATTGCCCGACTTCACCCA-3′	periplasmic serine peptidase Do/DeqQ	KGA03014
Tryp_R	5′–TATACTCGAGTCACTTGTCGCTGTCGGCACGCATG-3′		
CmClp_F	5′-TATCCATGGTAAACGACTTCGACATCAAGAATGCT-3′	ATP-dependent caseinolytic protease Clp	KGA03297
CmClp_R	5′-TATAGAGCTCTCACTCCACGTCGGGACGGCGTTCC-3′		
X-PhoN_F	5′-TTAACCATGGCAGAGATCAAGAATGTCATTCTGAT-3′	alkaline phosphatase PhoA	DQ435608
CmAP_R	5′-TTAAGAATTCCTTCGCTACCACTGTCTTCAGATACTGTCC-3′		

* The molecular markers are the enzymes, predicted according to the structural classification of the National Center for Biotechnology Information (NCBI) Conserved Domain Database [23]; ** the reference genes’ IDs are from the whole genome sequence annotation of the *C. amphilecti* KMM 296 (GenBank ID: JQJA00000000.1).

## Data Availability

The data presented in this study are available on request from the corresponding author.

## Supplementary Materials

The following are available online, Table S1: Similarity calculation of 16S rRNA gene sequences of *Cobetia* isolates by EzBioCloud 16S database, Table S2: Similarity calculation of 16S RNA gene sequence of the type strain *Cobetia marina* LMG 2217T, Table S3: Similarity calculation of 16S RNA gene sequence of the strain *Cobetia amphilecti* KMM 296, Table S4: Comparative analysis of coding DNA sequences of *Cobetia* sp. 2AS1 (KMM 7005) and *C. amphilecti* KMM 296.

## Sample Availability

Samples of the isolates and DNA are available from the authors.

## References

[1] Cobet A.B., Wirsen C.J., Jones G.E. (1970). The effect of nickel on a marine bacterium, *Arthrobacter marinus* sp.nov. J. Gen. Microbiol..

[2] Baumann L., Baumann P., Mandel M., Allen R.D. (1972). Taxonomy of aerobic marine eubacteria. J. Bacteriol..

[3] Baumann L., Bowditch R.D., Baumann P. (1983). Description of *Deleya* gen. nov. created to accommodate the marine species *Alcaligenes aestus*, *A. pacificus*, *A. cupidus*, *A. venustus*, and *Pseudomonas marina*. Int. J. Syst. Bacteriol..

[4] Dobson S.J., Franzmann P.D. (1996). Unification of the genera *Deleya* (Baumann et al. 1983), *Halomonas* (Vreeland et al. 1980), and *Halovibrio* (Fendrich 1988) and the species *Paracoccus halodenitrificans* (Robinson and Gibbons 1952) into a single genus, *Halomonas*, and placement of the genus *Zymobacter* in the family *Halomonadaceae*. Int. J. Syst. Bacteriol..

[5] Arahal D.R., Castillo A.M., Ludwig W., Schleifer K.H., Ventosa A. (2002). Proposal of *Cobetia marina* gen. nov., comb. nov., within the family *Halomonadaceae*, to include the species *Halomonas marina*. Syst. Appl. Microbiol..

[6] Kim M.S., Roh S.W., Bae J.W. (2010). *Cobetia crustatorum* sp. nov., a novel slightly halophilic bacterium isolated from traditional fermented seafood in Korea. Int. J. Syst. Evol. Microbiol..

[7] Romanenko L.A., Tanaka N., Svetashev V.I., Falsen E. (2013). Description of *Cobetia amphilecti* sp. nov., *Cobetia litoralis* sp. nov. and *Cobetia pacifica* sp. nov., classification of *Halomonas halodurans* as a later heterotypic synonym of *Cobetia marina* and emended descriptions of the genus *Cobetia* and *Cobetia marina*. Int. J. Syst. Evol. Microbiol..

[8] Tang X., Xu K., Han X., Mo Z., Mao Y. (2018). Complete genome of *Cobetia marina* JCM 21022T and phylogenomic analysis of the family *Halomonadaceae*. J. Oceanol. Limnol..

[9] Balabanova L.A., Golotin V.A., Kovalchuk S.N., Babii A.V., Shevchenko L.S., Son O.M., Kosovsky G.Y., Rasskazov V.A. (2016). The Genome of the marine bacterium *Cobetia marina* KMM 296 isolated from the mussel *Crenomytilus grayanus* (Dunker, 1853). Russ. J. Mar. Biol..

[10] Cheng W., Yan X., Xiao J., Chen Y., Chen M., Jin J., Bai Y., Wang Q., Liao Z., Chen Q. (2020). Isolation, identification, and whole genome sequence analysis of the alginate-degrading bacterium *Cobetia* sp. cqz5-12. Sci. Rep..

[11] Guo P., Cao B., Qiu X., Lin J. (2018). Draft genome sequence of the crude oil-degrading and biosurfactant-producing strain *Cobetia* sp. QF-1. Genome Announc..

[12] Balabanova L., Podvolotskaya A., Slepchenko L., Eliseikina M., Noskova Y., Nedashkovskaya O., Son O., Tekutyeva L., Rasskazov V. (2017). Nucleolytic enzymes from the marine bacterium *Cobetia amphilecti* KMM 296 with antibiofilm activity and biopreservative effect on meat products. Food Control..

[13] Moriya H., Takita Y., Matsumoto A., Yamahata Y., Nishimukai M., Miyazaki M., Shimoi H., Kawai S.J., Yamada M. (2020). *Cobetia* sp. bacteria, which are capable of utilizing alginate or waste *Laminaria* sp. for poly(3-hydroxybutyrate) synthesis, isolated from a marine environment. Front. Bioeng. Biotechnol..

[14] Mei R., Zhou M., Xu L., Zhang Y., Su X. (2019). Characterization of a pH-tolerant strain *Cobetia* sp. SASS1 and its phenol degradation performance under salinity condition. Front. Microbiol..

[15] Coenye T., Vandamme P. (2003). Intragenomic heterogeneity between multiple 16S ribosomal RNA operons in sequenced bacterial genomes. FEMS Microbiol. Lett..

[16] Weisburg W.G., Barns S.M., Pelletier D.A., Lane D.J. (1991). 16S ribosomal DNA amplification for phylogenetic study. J. Bacteriol..

[17] Franco-Duarte R., Černáková L., Kadam S., Kaushik K.S., Salehi B., Bevilacqua A., Corbo M.R., Antolak H., Dybka-Stępień K., Leszczewicz M. (2019). Advances in chemical and biological methods to identify microorganisms-from past to present. Microorganisms.

[18] Liu W., Li L., Khan M.A., Zhu F. (2012). Popular molecular markers in bacteria. Mol. Gen. Mikrobiol. Virusol..

[19] Ashok Kumar J., Vinaya Kumar K., Avunje S., Akhil V., Ashok S., Kumar S., Sivamani B., Grover M., Rai A., Alavandi S.V. (2020). Phylogenetic relationship among brackishwater *Vibrio* Species. Evol. Bioinform. Online..

[20] Ivanova E., Christen R., Sawabe T., Alexeeva Y., Lysenko A., Chelomin V., Mikhailov V. (2005). Presence of ecophysiologically diverse populations within *Cobetia marina* strains isolated from marine invertebrate, algae and the environments. Microbes Environ..

[21] Balabanova L., Nedashkovskaya O., Podvolotskaya A., Slepchenko L., Golotin V., Belik A., Shevchenko L., Son O., Rasskazov V. (2016). Data supporting functional diversity of the marine bacterium *Cobetia amphilecti* KMM 296. Data Brief.

[22] Yoon S.H., Ha S.M., Kwon S., Lim J., Kim Y., Seo H., Chun J. (2017). Introducing EzBioCloud: A taxonomically united database of 16S rRNA gene sequences and whole-genome assemblies. Int. J. Syst. Evol. Microbiol..

[23] Lu S., Wang J., Chitsaz F., Derbyshire M.K., Geer R.C., Gonzales N.R., Gwadz M., Hurwitz D.I., Marchler G.H., Song J.S. (2020). CDD/SPARCLE: The conserved domain database in 2020. Nucleic Acids Res..

[24] Plisova E.Y., Balabanova L.A., Ivanova E.P., Kozhemyako V.B., Mikhailov V.V., Agafonova E.V., Rasskazov V.A. (2005). A highly active alkaline phosphatase from the marine bacterium *Cobetia*. Mar. Biotechnol..

[25] Noskova Y., Likhatskaya G., Terentieva N., Son O., Tekutyeva L., Balabanova L. (2019). A novel alkaline phosphatase/phosphodiesterase, CamPhoD, from marine bacterium *Cobetia amphilecti* KMM 296. Mar. Drugs..

[26] Jiang N., Tu Z., Zhang Y., Li J., Feng Y., Yang N., Sang X., Chen Q. (2018). Identification and characterization of DNA endonucleases in Plasmodium falciparum 3D7 clone. Malar. J..

[27] Zhang Y., Chen T., Zheng W., Li Z.H., Ying R.F., Tang Z.X., Shi L.E. (2018). Active sites and thermostability of a non-specific nuclease from *Yersinia enterocolitica* subsp. palearctica by site-directed mutagenesis. Biotechnol. Biotechnol. Equip..

[28] Liu Q., Wang X., Qin J., Cheng S., Yeo W.-S., He L., Ma X., Liu X., Li M., Bae T. (2017). 2017 The ATP-dependent protease ClpP inhibits biofilm formation by regulating Agr and Cell wall hydrolase Sle1 in *Staphylococcus aureus*. Front. Cell. Infect. Microbiol..

[29] Sutto-Ortiz P., Camacho-Ruiz M., Kirchmayr M.R., Camacho-Ruiz R.M., Mateos-Díaz J.C., Noiriel A., Carrière F., Abousalham A., Rodríguez J.A. (2017). Screening of phospholipase A activity and its production by new actinomycete strains cultivated by solid-state fermentation. PeerJ.

[30] Golotin V., Balabanova L., Likhatskaya G., Rasskazov V. (2015). Recombinant production and characterization of a highly active alkaline phosphatase from marine bacterium *Cobetia marina*. Mar. Biotechnol. (NY).

[31] Overbeek R., Olson R., Pusch G.D., Olsen G.J., Davis J.J., Disz T., Edwards R.A., Gerdes S., Parrello B., Shukla M. (2014). The SEED and the Rapid Annotation of microbial genomes using Subsystems Technology (RAST). Nucleic Acids Res..

[32] Vos M. (2011). A species concept for bacteria based on adaptive divergence. Trends Microbiol..

[33] Rader B.A., Kremer N., Apicella M.A., Goldman W.E., McFall-Ngai M.J. (2012). Modulation of symbiont lipid A signaling by host alkaline phosphatases in the squid-vibrio symbiosis. mBio..

[34] Xie M.Ø., Olderøy M., Zhang Z., Andreassen J.-P., Strandd B.L., Sikorski P. (2012). Biocomposites prepared by alkaline phosphatase mediated mineralization of alginate microbeads. RSC Adv..

[35] Ivanova E.P., Mikhailo V.V., Plisova E.J., Balabanova L.A., Svetashev V.V., Vysockyi M.V., Stepanenko V.I., Rasskazov V.A. (1994). Characterization of the marine bacterium *Deleya marina* producing highly active alkaline phosphatase and associated with the mussel *Crenomytilus grayanus*. Russ. J. Mar. Biol..

