# Supplementary material for: Are the Closely Related Cobetia Strains of Different Species?
Source: Molecules. 2021 Jan 28;26(3):690. doi: 10.3390/molecules26030690 (PMC7865433; doi:10.3390/molecules26030690)

**Table S1.** Similarity calculation of 16S rRNA gene sequences of *Cobetia* isolates by EzBioCloud 16S database


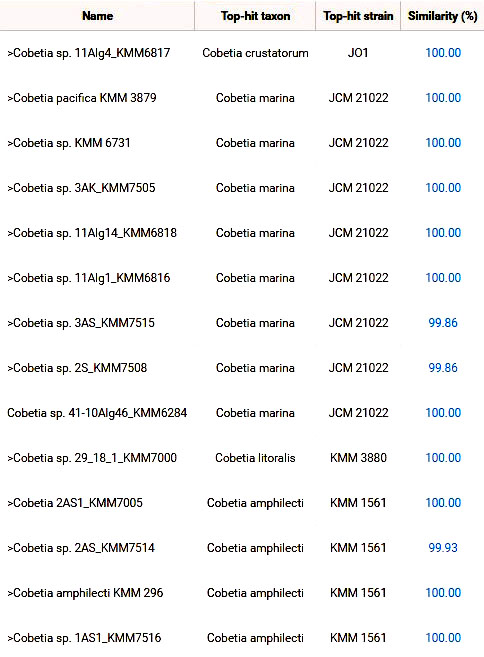


**Table S2.** Similarity calculation of 16S RNA gene sequence of the type strain *Cobetia marina* LMG 2217^T^


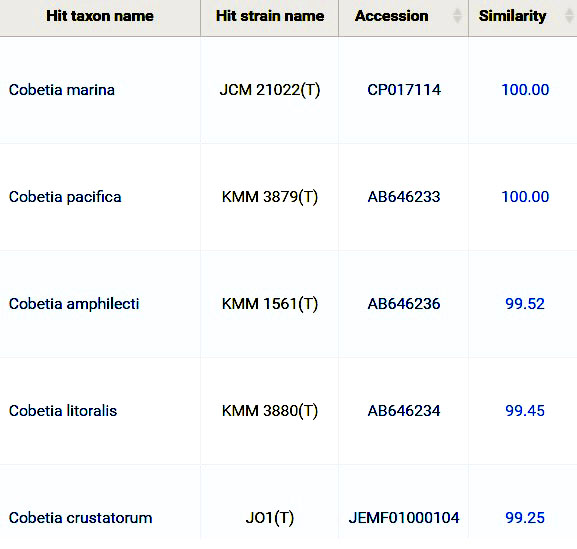


**Table S3.** Similarity calculation of 16S RNA gene sequence of the strain *Cobetia* *amphilecti* KMM 296


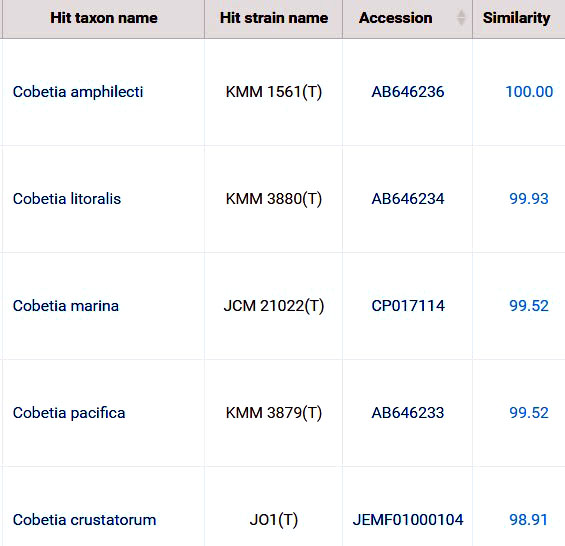

Supplement: Supplementary file 1 [file molecules-26-00690-s001.zip › molecules-1062096-supplementary-final/Table S1-S3.docx]
